# NbNAC42 and NbZFP3 Transcription Factors Regulate the Virus Inducible *NbAGO5* Promoter in *Nicotiana benthamiana*

**DOI:** 10.3389/fpls.2022.924482

**Published:** 2022-06-23

**Authors:** Yuan-Dun Ke, Ying-Wen Huang, Kotapati Kasi Viswanath, Chung-Chi Hu, Chuan-Ming Yeh, Nobutaka Mitsuda, Na-Sheng Lin, Yau-Heiu Hsu

**Affiliations:** ^1^Graduate Institute of Biotechnology, National Chung Hsing University, Taichung, Taiwan; ^2^Advanced Plant Biotechnology Center, National Chung Hsing University, Taichung, Taiwan; ^3^Institute of Molecular Biology, National Chung Hsing University, Taichung, Taiwan; ^4^Bioproduction Research Institute, National Institute of Advanced Industrial Science and Technology (AIST), Tsukuba, Japan; ^5^Institute of Plant and Microbial Biology, Academia Sinica, Taipei City, Taiwan

**Keywords:** argonaute, AGO5, BaMV, TGBp1, potexvirus, NAC, zinc finger protein (ZFP)

## Abstract

Plant argonautes (AGOs) play important roles in the defense responses against viruses. The expression of *Nicotiana benthamiana AGO5* gene (*NbAGO5*) is highly induced by *Bamboo mosaic virus* (BaMV) infection; however, the underlying mechanisms remain elusive. In this study, we have analyzed the potential promoter activities of *NbAGO5* and its interactions with viral proteins by using a 2,000 bp fragment, designated as PN1, upstream to the translation initiation of *NbAGO5.* PN1 and seven serial 5′-deletion mutants (PN2–PN8) were fused with a β-glucuronidase (GUS) reporter and introduced into the *N. benthamiana* genome by *Agrobacterium*-mediated transformation for further characterization. It was found that PN4-GUS transgenic plants were able to drive strong GUS expression in the whole plant. In the virus infection tests, the GUS activity was strongly induced in PN4-GUS transgenic plants after being challenged with potexviruses. Infiltration of the transgenic plants individually with BaMV coat protein (CP) or triple gene block protein 1 (TGBp1) revealed that only TGBp1 was crucial for inducing the *NbAGO5* promoter. To identify the factors responsible for controlling the activity of the *NbAGO5* promoter, we employed yeast one-hybrid screening on a transcription factor cDNA library. The result showed that NbNAC42 and NbZFP3 could directly bind the 704 bp promoter regions of *NbAGO5*. By using overexpressing and virus-induced gene silencing techniques, we found that NbNAC42 and NbZFP3 regulated and downregulated, respectively, the expression of the *NbAGO5* gene. Upon virus infection, NbNAC42 played an important role in regulating the expression of NbAGO5. Together, these results provide new insights into the modulation of the defense mechanism of *N. benthamiana* against viruses. This virus inducible promoter could be an ideal candidate to drive the target gene expression that could improve the anti-virus abilities of crops in the future.

## Introduction

*Nicotiana benthamiana* can be infected by diverse plant viruses, making it a popular model host in many aspects of plant virology (Goodin et al., 2008). A variety of host factors in *N. benthamiana* involved in the infection processes of viruses have been identified in previous studies (Huang et al., 2012, 2017b, 2019, 2021). However, the defense machineries of *N. benthamiana* against viruses remain relatively unexplored. Among the few well-studied virus resistance mechanisms, it has been shown that plant viruses may induce a potent and specific antiviral RNA silencing host response in which argonautes (AGOs) play a central role (Carbonell and Carrington, 2015). Antiviral AGOs may associate with virus-derived small RNAs (vsiRNAs) to repress the expression of the cognate viral RNAs or DNAs, or with endogenous small RNAs (sRNAs) to regulate host gene expression and promote antiviral defense (Baulcombe, 1999; Carbonell and Carrington, 2015; Csorba et al., 2015; Alazem et al., 2017). The gene silencing mechanism, mainly through the interactions of AGOs with sRNAs, plays an important role in the regulation of gene expression in plants (Meister, 2013), and is an integral part of the essential innate immunity to fight against both RNA and DNA viruses in plants.

Both mono- and dicotyledonous plants encode several AGO proteins that can be categorized into three distinct clades: AGO 1/5/10, AGO 2/3/7, and AGO 4/6/8/9 based on phylogenetic and functional relationships (Vaucheret, 2008; Fang and Qi, 2016). Among them, AGO 1/5/10 clade is mainly involved in anti-viral activities. Several virus-resistant AGO members have been identified in *N. benthamiana*. NbAGO1 has been reported to be required for resistance to *Tomato ring spot virus* (ToRSV) (Ghoshal and Sanfacon, 2014), *Tomato bushy stunt virus* (TBSV), and *Cymbidium ringspot virus* (CymRSV) (Gursinsky et al., 2015), *Turnip crinkle virus* (TCV) (Ludman et al., 2017), and *Bamboo mosaic virus* (BaMV) (Huang et al., 2019); NbAGO2 has been shown to play a role in the resistance against TBSV, *Tobacco mosaic virus* (TMV) (Diao et al., 2019), *Potato virus X* (PVX), *Turnip mosaic virus* (TuMV), and TCV (Fatyol et al., 2016; Alazem et al., 2017); NbAGO4 is involved in PVX resistance (Bhattacharjee et al., 2009); NbAGO5 is also able to bind *Cucumber mosaic virus* (CMV) vsiRNAs (Takeda et al., 2008), indicating a role in antiviral defense. It has also been shown that AGO5 is induced by PVX and cooperates with AGO2 to restrict viral infection (Brosseau and Moffett, 2015); NbAGO7 is related to resistance against *Foxtail mosaic virus* (FoMV), *Sunn-hemp mosaic virus* (SHMV), and TBSV (Odokonyero et al., 2017). The presence of specialized proteins known as viral suppressors of RNA silencing (VSRs) in viral genomes is one of the strongest indications of the significance of RNA silencing as an antiviral response (Csorba et al., 2015). The successful infection of a given host by a virus generally indicates that the virus has overcome the RNA silencing mechanisms of the host, possibly *via* the action of its VSR. Most VSRs are multifunctional proteins with a secondary role of VSR activity because many viral proteins are involved in the replication, movement, and/or encapsidation of viruses. VSRs have different modes of action, including sequestration of sRNAs and proteins necessary for the onset of RNA silencing and the propagation of signals. VSRs may also exert their functions by shifting the balances among different defense-related host factors. Previous studies have shown that NbAGO10 could facilitate the accumulation of viruses mainly because P28 (TGBp1) in BaMV promoted the expression of NbAGO10, which competes with NbAGO1 for vsiRNA and leads to a decrease in the antiviral ability of AGO1 (Huang et al., 2019). Thus, specific AGOs may be recruited in the defense responses against different viruses. However, the underlying mechanism for the modulation of specific AGO gene expression by specific viral factors awaits further exploration.

In plants, the crosstalk among phytohormones is crucial in the responses to multiple biotic and abiotic stimuli, including the infection of viruses. It has been shown that the promoters of almost all AGO-encoding genes contain motifs involved in the binding by salicylic acid (SA) or abscisic acid (ABA)-responsive transcription factors (TFs) (Alazem et al., 2019). During virus infection in plants, several AGO-encoding genes are upregulated (Bai et al., 2012; Alazem et al., 2017; Paudel et al., 2018; Diao et al., 2019; Sheng et al., 2019). In addition to phytohormones, plants have evolved a number of molecular mechanisms for dealing with stress, including the augmentation or reduction of the effects of TFs on specific target genes. In the responses to abiotic and biotic stressors, plant TFs may operate as nodes in a regulatory network. Many members of diverse families of TFs, including MYB (Yang and Klessig, 1996; Yang et al., 2020), WRKY (Park et al., 2006; Chen et al., 2013), bZIP (Gaguancela et al., 2016; Gayral et al., 2020), AP2/ERF (Huang et al., 2016), and NAC (Ren et al., 2000; Selth et al., 2005; Donze et al., 2014; Huang et al., 2017a; Sun et al., 2019), have been demonstrated to be involved in the responses to viral stimuli. In addition, TFs with “zinc finger” domains (zinc finger protein, ZFP) known for their finger-like structure and the ability to bind Zn^+2^ ions have also been implicated in response to viral infections (Guo et al., 2004; Huh et al., 2011). However, it remains to be explored to what extent phytohormones and TFs affect the expression and function of AGOs during virus infection.

In *Arabidopsis thaliana*, AGO5 is normally expressed mostly in flowers and other reproductive tissues, but the *AGO5* gene is systemically activated in leaves after the infection of PVX or *Plantago asiatica mosaic virus*, implicating that AGO5 plays an important role in limiting the spread of systemic viruses (Brosseau and Moffett, 2015). In orchids, the AGO5 family proteins play an important part in the defense against the *Cymbidium mosaic virus* (CymMV) and the *Odontoglossum ringspot virus* (ORSV) (Kuo et al., 2021). Interestingly, we have found that *NbAGO5* is strongly induced by BaMV infection in *N. benthamiana*, which provides a feasible system to elucidate the underlying mechanisms involved in antiviral activities. Thus, the objective of this study was to identify the factors which trigger the expression of NbAGO5, by investigating the *NbAGO5* promoter activities in transgenic plants. In addition, we also aimed at identifying the TFs that affect the expression of *NbAGO5* promoter by screening the Arabidopsis TFs cDNA library using yeast one-hybrid assay.

## Materials and Methods

### Plant Cultivation Conditions

Unless otherwise stated, all *N. benthamiana* plants were grown in a growth room maintained at 26 ± 1°C under a photoperiod of 16 h-light/8 h-dark.

### Mechanical Inoculation of Bamboo Mosaic Virus

The fourth leaves from the bottom to the top along the stem of seedlings of the 30-day-old *N. benthamiana* were mechanically inoculated with 0.5 μg of the BaMV severe strain (BaMV-S) virion (Lin et al., 1994). The relative expression of Argonautes mRNAs in inoculated leaves was determined using real-time quantitative reverse transcription PCR (qRT-PCR), with actin serving as an internal control.

### Construction of Viral Infectious Clones and Inoculation Assay

The infectious constructs, based on the plasmid backbone pKn (Prasanth et al., 2011), of the viruses used in this study included pKB (Liou et al., 2014) for BaMV, pKP (Huang et al., 2012) for PVX, pKF (Huang et al., 2020) for FoMV, pKCy1 (Huang et al., 2020) for CymMV, and pKT for TMV. For the construction of pKT, the cDNA of TMV was reverse transcribed with primer *Kpn*I_TMV-R (Supplementary Table 1) from TMV RNA that was purified from TMV_Taiwan isolates. After amplification with primer TMV_5′-F (Supplementary Table 1), the PCR products were cloned into pCass (Ding et al., 1995) at the appropriate restriction sites to generate pCT, and then sub-cloned into *Sbf*I and *Kpn*I restriction sites of pKn to generate pKT construct.

For the construction of CMV infectious clones, the cDNAs of CMV_NT9 RNA1, RNA2, and RNA3 were reverse transcribed with primers, *Sac*I_CMV1a_R, *Hpa*I-*Kpn*I_CMV2a_R, and *Sac*I_CMV3a_R, respectively (Supplementary Table 1). After amplification with forward primers for CMV RNA1, RNA2, and RNA3, *Bam*HI/*Sac*I_CMV1a, *Bam*HI/*Hpa*I-*Kpn*I_CMV2a, and *Bam*HI/*Sac*I_CMV3a, respectively, the PCR products were cloned into pEpyon-GFP (Huang et al., 2017b) at the appropriate restriction sites to generate constructs, pECMV1, pECMV2, and pECMV3.

For the construction of transient expression clones of BaMV-CP and -TGBp1, the corresponding coding regions were amplified by PCR using pKB as the template with the primers, XbaI_BaCP-F and *Sac*I_BaCP-R for BaMV-CP, *Xba*I_BaTGBp1-F and *Sac*I_BaTGBp1-R for BaMV-TGBp1 (Supplementary Table 1), and cloned into pEpyon-GFP at the *Bam*HI and *Eco*RI restriction sites to generate pEBaCP and pEBaTGBp1 constructs, respectively. The infectious constructs and transient expression plasmids were introduced into *Agrobacterium tumefaciens* strain GV3850 individually by electroporation. The third, fourth, and fifth leaves from the bottom to the top along the stem of seedlings of the 30-day-old *N. benthamiana* were infiltrated with *A. tumefaciens* harboring the above constructs as described previously (Huang et al., 2012).

### Real-Time Quantitative Reverse Transcription

Total RNA was extracted from leaf tissues using TriPure Isolation Reagent (Roche Life Science, Mannheim, Germany) according to the manufacturer’s instructions. First-strand cDNAs were synthesized using GoScript Reverse Transcriptase (Promega, Madison, WI, United States) with the oligo dT(18) primer. The qRT-PCR analyzes of *NbAGO5* or TFs mRNA levels were performed with a TOptical Gradient 96 Real-Time PCR Thermal Cycler (Biometra, Göttingen, Germany) in reactions containing 3 μl of two-fold-diluted cDNAs as templates specific primers (Supplementary Table 1), and the KAPA SYBR^®^ FAST qPCR master mix (Kapa Biosystems, Wilmington, MA, United States). The internal control was actin, and the experiments were performed in triplicate.

### Isolation of *NbAGO5* Promoter Sequence

Primers were designed based on the 2 kb region upstream to the ATG start codon of *NbAGO5* gene in *N. benthamiana* genome (Nbv6.1trP59647) downloaded from the QUT database^1^. The putative *NbAGO5* promoter region was amplified by PCR from *N. benthamiana* genomic DNA with PNP1 primers (Supplementary Table 1). The amplicon containing the 2,000 bp putative promoter region, designated PN1, of the *NbAGO5* gene was cloned and sequenced.

### *In silico* Analysis of *NbAGO5* Promoter Sequence

To predict the potential interacting transcription factors, the *cis*-regulatory elements of *NbAGO5* promoter were analyzed using PlantCARE web server^2^ (Lescot et al., 2002).

### Construction of Promoter: β-Glucuronidase Fusion and Deletion Vectors

**FIGURE 1:**
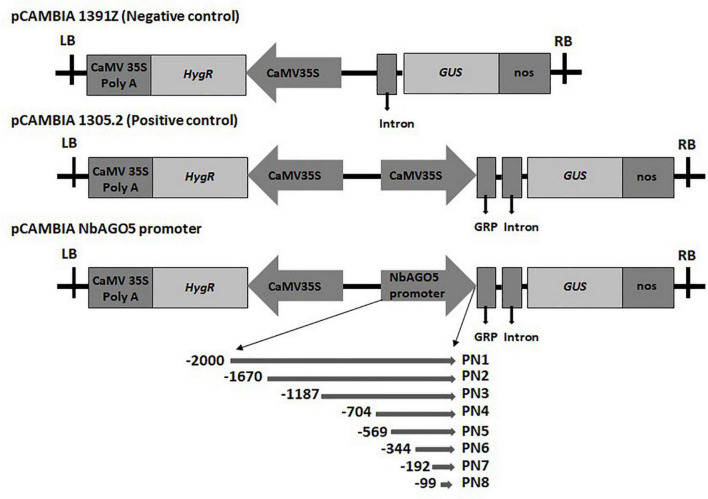
Schematic diagrams of the truncated fragments of different deleted versions fused with the GUS reporter gene. The pCAMBIA1391Z vector acts as a negative control that does not have a promoter to drive GUS. The pCAMBIA1301 vector was used as a positive control, and the pCAMBIA *NbAGO5* promoter constructs with different lengths of *NbAGO5* promoter was created by replacing the 35S promoter (A). LB, left border; CaMV 35S PolyA, cauliflower mosaic virus 35S terminator; *HygR*, hygromycin resistance gene; 35S promoter, cauliflower mosaic virus 35S promoter; *GRP*, glycine-rich signal peptide sequence; GUS, β-glucuronidase reporter gene; Nos, nopaline synthase terminator; RB, right border.

The initial division of the putative “full-length” promoter region was based on the presence of two restriction sites, *Hin*dIII and *Xba*I, which allows for a convenient sub-cloning process. To further fine-map the functional promoter region, PN2 and PN5 were further divided into shorter fragments based on the design of proper primer sequences for PCR amplification. Five serial 5′-deletion fragments (ranging from nucleotide positions −1,187, −704, −344, −192, and −99 to position −1, with the translation initiation site designated as “+1”; Figure 1) were amplified by PCR from the PN1 region with specific oligonucleotide primers (Supplementary Table 1). For the generation of *NbAGO5*:GUS constructs, the PCR products were subsequently constructed into the pCAMBIA1305.2 vector (Marker Gene Technologies, Ipswich, England) at the *Xba*I or *Hin*dIII*/Nco*I restriction sites. The resulting constructs were confirmed by sequencing and used for *N. benthamiana* stable transformation.

### Culture and Stable Transformation of *Nicotiana benthamiana*

The *NbAGO5*:GUS plasmids were introduced into *A. tumefaciens* strain Agro 3,850 individually by electroporation (Kotnik, 2013). The *A. tumefaciens*-mediated transformation procedures were followed as previously described (Horsch et al., 1985). Regeneration of the transformants was performed using the method as described (van Tunen et al., 1989). The T_0_ positive transformants were screened by hygromycin resistance (Clough and Bent, 1998). The homozygous transgenic lines of T_3_ generation were chosen for subsequent investigations *via* segregation ratio analysis.

### Hormone Treatments in Transgenic *Nicotiana benthamiana*

Seeds of transgenic plants were germinated and grown in liquid MS medium at 20°C under a 16/8 h photoperiod in 6-well plates. When seedlings were 6 days old, the liquid MS medium was replaced with fresh MS containing ABA (10 μM), SA (50 μM), Methyl jasmonate (MeJA) (50 μM), or mock (0.5% EtOH in ddH_2_O) (Alazem et al., 2019). Two days after treatment, 10 seedlings from each line were infiltrated with the above viral infectious constructs, and the leaves were collected at 2 days post infiltration (dpi) and analyzed for GUS activities.

### Analysis of Virus-Induced Gene Expression in Transgenic *Nicotiana benthamiana*

For analyzing stress responses caused by viruses, *A. tumefaciens* cells harboring infectious clones of potexviruses (BaMV, PVX, FoMV, and CymMV) were infiltrated (hereafter referred to as agroinfiltrated) into transgenic plants as follows. The *A. tumefaciens* cultures were collected by centrifugation and resuspended in infiltration buffer (10 mM MES buffer, pH 5.5, and 10 mM MgCl_2_), adjusted to OD_600_ = 0.5, and infiltrated into the leaves of each test plant through a needleless syringe. The third, fourth, and fifth leaves, from the bottom to the top of the 30-day-old *N. benthamiana* seedlings, were agroinfiltrated. The leaves were harvested at 3 dpi and subjected to a GUS activity assay.

### Histochemical β-Glucuronidase Staining and β-Glucuronidase Activity Quantification

Total proteins from infiltrated plants were extracted and quantified using the method described previously (Bradford, 1976). Histochemical GUS staining and GUS fluorometric analyzes were performed by following standard methods (Jefferson et al., 1987).

### Yeast One-Hybrid Screening at *Arabidopsis* Transcription Factors Library

To identify the TFs that could interact with the *NbAGO5* promoter, a systematic screening of the Y1H TFs library, which contains approximately 1,350 *Arabidopsis* TFs (Mitsuda et al., 2010) was undertaken in yeast strain YM4271.

For Y1H assays, the prey protein was fused to the activation domain (AD) of the yeast Gal4 transcription factor. The positive interaction between the prey protein and the bait DNA sequence (putative AGO5 promoter region) caused HIS3 reporter gene expression in *Saccharomyces cerevisiae*. Preparation of yeast competent cell, yeast transformation, and selection of interaction were carried out according to the user manual of the manufacturer (Invitrogen, Thermo Fisher, Taipei, Taiwan). The bait and prey-designated plasmids were combined as indicated and were transformed simultaneously into a Y187 cell and plated on an SD/-Leu-Ura for selection of successfully co-transformed cells. Three colonies were selected at random, dissolved in water, and plated on an SD/-Leu-Ura-His for the selection of a specific interaction.

For the construction of bait plasmid pHISi-PN4, the 704 bp promoter region of *NbAGO5* (PN4) was amplified from the genomic DNA of *N. benthamiana* by using appropriate primers (Supplementary Table 1) with the restriction endonuclease sites of *Eco*RI and *Sac*I, cloned in the vector pHisi to give pHisi–PN4, and integrated into the yeast genome Y187. The Y1H assay was carried out as described previously (Mitsuda et al., 2010). In the screenings of *Arabidopsis* TFs library, the degree of positive interaction between a prey TF and the bait sequence was scored between 0 and 3 in each screening according to the yeast growth status under selective media so that each TF has its respective interaction strength.

### Corresponding Transcription Factors in *Nicotiana benthamiana* Transcriptome

The above Y1H screening generated candidate *A. thaliana* TFs with high binding affinity to the putative promoter region of *NbAGO5*. To identify the corresponding TFs in *N. benthamiana* genome, the sequences of the candidate *A. thaliana* TFs were used as the query to search for the corresponding TF homologs in *N. benthamiana* transcriptome database.^3^ Following the identification of the corresponding TFs in *N. benthamiana*, oligonucleotide primers were designed (Supplementary Table 1) to amplify the respective *N. benthamiana* TFs by PCR. The PCR products were then cloned into pGADT7 (Mitsuda et al., 2010), pEpyon, and pTRV2 vector (Ratcliff et al., 2001) at *Eco*RI and *Bam*HI restriction sites for further Y1H, over-expression, and virus-induced gene silencing (VIGS) analyzes, respectively.

### Yeast One-Hybrid Assay

To verify the interactions between the putative AGO5 promoter region and candidate *N. benthamiana* TFs or BaMV encoded proteins, yeast one-hybrid assays were performed. The coding regions of BaMV-CP and -TGBp1 and candidate *N. benthamiana* TFs were amplified by PCR with specific primer pairs (Supplementary Table 1) and cloned into the respective restriction sites of pGADT7 vector to give the prey constructs pGADT7-CP, pGADT7-TGBp1, pGADT7-NbNAC42, and pGADT7-NbZFP3. The plasmids and pHisi–PN4 were transformed into the yeast genome Y187. The transformants were grown on SD minimal medium lacking Leu, Ura, and His but containing 20 mM 3-AT. The cell concentration was adjusted to OD_600_ = 1 and serial dilutions were spotted on plates and grown for 3 days at 28°C.

### Transient Expression and Virus-Induced Gene Silencing of Candidate Transcription Factors in *Nicotiana benthamiana*

The coding regions of candidate TFs were amplified with specific primers (Supplementary Table 1) and cloned into transient expression vector pEpyon. The pEpyon-based constructs of candidate TFs were introduced into *A. tumefaciens* strain GV3850 individually by electroporation. The *A. tumefaciens* cultures were collected by centrifugation and resuspended in infiltration buffer (10 mM MES buffer, pH 5.5 and 10 mM MgCl_2_), adjusted to OD_600_ = 0.5, and infiltrated into the leaves of each test plant through needleless syringes. The leaves were harvested at 3 dpi and subjected to GUS activity assay and western blot as described previously (Huang et al., 2019).

To transiently knockdown the expression of the candidate TFs, the VIGS technique (Ratcliff et al., 2001) was employed. The target sequences of candidate TFs were amplified from *N. benthamiana* genomic DNA with the oligonucleotide primers (Supplementary Table 1), which were designed based on the sequences of specific TFs downloaded from the SGN database (Solanaceae Genomics Network^4^). The PCR products were subsequently constructed into the pTRV2 vector at the corresponding restriction sites. For knockdown experiments, the pTRV1- and pTRV2-based constructs were electroporated into the *A. tumefaciens* strain C58C1 and infiltrated into test plants as described previously (Huang et al., 2012).

### Statistical Analysis

All inoculation and Y1H experiments were performed with at least three biological replicates. The data were analyzed using a basic statistic tool in EXCEL 2016 for Windows 10 (Microsoft), including ANOVA. Statistical differences between means of groups were determined by Student’s *t*-test, with *P*-value ≤ 0.05; ≤0.01, or ≤0.001 as statistically significant or highly significant (denoted by “*”; “^**^” or “^***^”), respectively.

## Results

### *NbAGOs* mRNA Expression Profile After Bamboo Mosaic Virus Infection

**FIGURE 2:**
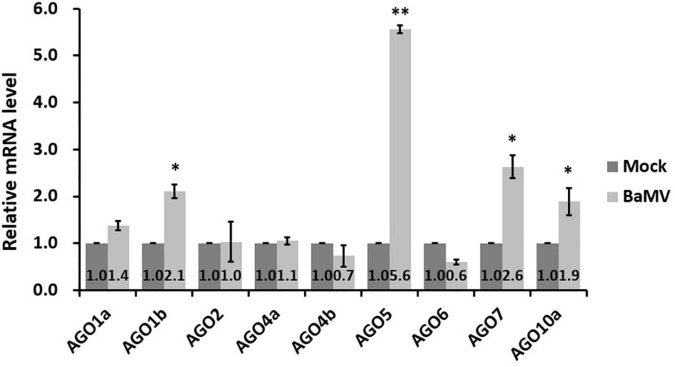
Realtime RT-PCR analyzes of Argonaute genes in *Bamboo mosaic virus* (BaMV)-inoculated *Nicotiana benthamiana*. *Nicotiana benthamiana* leaves were mechanically inoculated with 0.5 μg of BaMV virions and then collected at 5 dpi for analysis. Real-time quantitative reverse transcription PCR (qRT-PCR) was used to determine the relative expression of Argonautes mRNAs in inoculated leaves, with actin serving as an internal control. Each column represents the average of at least three replicates ± standard error (SE). Statistical significance was analyzed using two-tailed student’s *t*-tests; *p < 0.05; **P < 0.01.

The NbAGOs corresponding to the ten AtAGOs, including isoforms, have been identified in the *N. benthamiana* genome. There are seven members of NbAGOs: namely NbAGO1, NbAGO2, NbAGO4, NbAGO5, NbAGO6, NbAGO7, and NbAGO10. Two isoforms were found for NbAGO1, NbAGO4, and NbAGO10. To analyze the mRNA expression profiles of different NbAGOs in response to the BaMV infection, total RNAs from mock- and BaMV-infected *N. benthamiana* leaves were extracted at 5 dpi and the mRNA levels of NbAGOs were quantified by qRT-PCR. The result revealed that *NbAGO5* mRNA levels were 5.6-fold higher in BaMV-inoculated leaves than that in mock-inoculated leaves at 5 dpi (Figure 2), which was the largest increase of all NbAGOs. In addition, the expression levels of *NbAGO1b, NbAGO7*, and *NbAGO10a* were also significantly higher than those in plants inoculated with an empty vector. In contrast, the expression level of *NbAGO10b* was not detectable in leaves of mock-inoculated or BaMV-inoculated *N. benthamiana* and was not shown in Figure 2. Thus, *NbAGO5* and its putative promoter region were chosen as the main subjects of the following analyzes.

### Isolation and Sequence Analysis of the *NbAGO5* Promoter

Initially, the putative *NbAGO5* promoter region was amplified and cloned from genomic DNA using specific primer pairs designed for the 2,000-nucleotide fragment upstream to the start codon of *NbAGO5* gene, designated as PN1. Following sequence verification, the putative *NbAGO5* promoter region was used as the query to search against the PlantCARE database for the presence of possible *cis*-acting elements for transcription. The positions and descriptions of the identified *cis*-acting elements are shown in Supplementary Table 2. The results revealed that the 2,000 bp promoter sequence contains a number of TATA-box and CAAT-box core *cis*-acting elements. Some *cis*-acting elements for the perception of multiple environmental stimuli are also present, including eight types of light-responsive elements (G-box, I-box, MRE, TCT-motif, AE-box, GT1-motif, Sp1, and TCT-motif), 3 types of abscisic acid-responsive elements (ABRE, ABRE3a, and ABRE4), one of ethylene-responsive element (ERE), one of SA responsive element (TCA-element), two gibberellin-responsive elements (GARE-motif and P-box), one known element required for meristem expression (CAT-box), a *cis*-acting regulatory element essential for the anaerobic induction (ARE), and some elements (MYB) that have different responses in growth or environmental stress. Thus, the putative *NbAGO5* promoter region has the potential to perceive the stimuli associated with growth, development, phytohormones and biotic or abiotic stresses in plants.

### Mapping of the Core Region of *NbAGO5* Promoter in Transgenic *Nicotiana benthamiana* Through 5′ Serial Deletions

**FIGURE 3:**
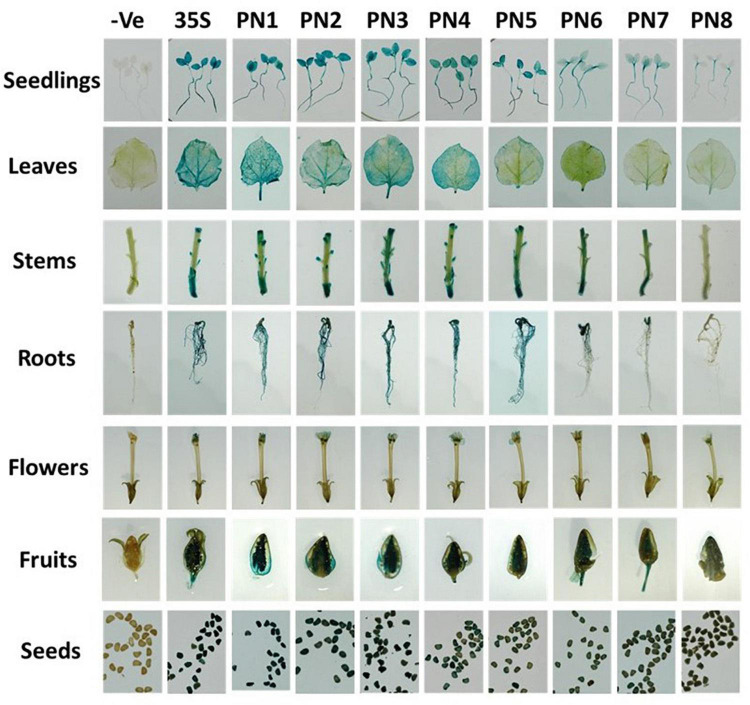
Histochemical GUS staining in tissues of T_3_ transgenic *N. benthamiana*. Plant organs from homozygous T_3_ generation transgenic lines were incubated in GUS staining solution for 24 h at 37°C. Then the samples were observed and photographed after decolorization.

To map the core region of *NbAGO5* promoter, 5′-serial deletions of PN1 fragment were prepared and fused with the GUS gene to produce reporter constructs used for generating transgenic *N. benthamiana*. Five to ten independent transgenic lines from each of the constructs (PN1–PN8, with CaMV35S promoter as a control) were screened in T_1_ generation by histochemical GUS staining. The homozygous lines in T_3_ generation were chosen for further analysis. The results of GUS staining showed that fragments PN1 to PN8 were all capable of driving GUS expression, but with obvious difference in their promoter activities in transgenic *N. benthamiana* (Figure 3). To analyze the expression profiles of transgenic *N. benthamiana* harboring GUS reporter gene driven by PN1 to PN8 or the CaMV 35S promoter under normal conditions, samples from different tissues, including the radicle of seeds from 10-day-old seedlings, roots, stems, and leaves of 70-day-old plants, flowers, fruits, and seeds from 90-day-old plants were subjected to GUS histochemical staining (Figure 3). GUS expressions driven by PN7 and PN8 were the lowest among all tissues. High GUS expression was detected in almost all tissues of plants harboring fragments PN1–PN6, including the seedlings, leaves, cotyledon, stem, petals, sepals, stigma, and seed. It is worth noting that, in leaves, PN4 seemed to possess the minimal length required for efficient promoter activity for driving GUS expression in transgenic plants, and thus might represent the core promoter region of *NbAGO5* gene.

**FIGURE 4:**
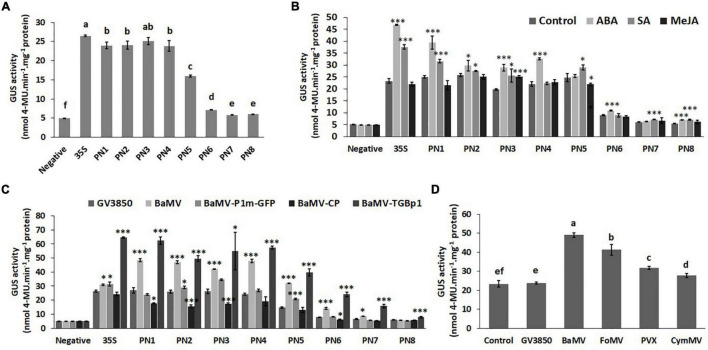
Promoter activity of *NbAGO5* under normal condition, hormone treatments, and challenge with the virus in Transgenic *N. benthamiana.* **(A)** The fluorometric GUS assays were performed with leaves of *transgenic N. benthamiana* harboring different constructs under normal conditions and **(B)** hormone treatments. **(C)** The GUS activities in the plants infiltrated with *A. tumefaciens* harboring infectious construct of BaMV or expression vector for BaMV-CP or BaMV-TGBp1 were analyzed at 3 dpi. **(D)** PN4 transgenic *N. benthamiana* plants were agro-infiltrated with infectious constructs of various potexviruses, including BaMV, PVX, FoMV, and CymMV. The GUS activities were then analyzed at 3 dpi. Each column represents the mean of at least three replicates ± standard error (SE). Statistical significance was analyzed using two-tailed student’s *t*-tests; *p < 0.05; ^**^p < 0.01; ^***^P < 0.001. The different lowercase letters above the bars indicate statistically significant differences among the means based on two-way ANOVA followed by Duncan’s multiple range test (*P* < 0.05).

To further quantify the promoter strengths of PN1–PN8 under normal conditions and to verify the core region of the *NbAGO5* promoter, fluorometric GUS assays were performed with leaves of transgenic *N. benthamiana* harboring different constructs. The result (Figure 4A) revealed that the promoter activities remained high with deletions up to position −704 (PN4), but significantly decreased for PN5–PN8, with PN7 and PN8 showing the lowest activities. The sharpest decrease was observed between PN5 and PN7, as compared to those of PN1–PN4, suggesting that the core NbAGO5 promoter region may reside between positions −704 and −192, relative to the initiation codon, whereas the region between −191 to −1 may still exhibit the basal level of promoter activity. Among them, the GUS expression driven by PN6 was obviously higher than those of PN7 and PN8. These results were in accordance with the result of the identification of *cis*-acting elements within PN1 (Supplementary Table 2), which showed that positions −191 to -1 bp do not contain TATA-box, whereas the region between −704 and -192 bp contains multiple *cis*-acting elements, such as TATA-box (Supplementary Table 2). Together, these results indicated that PN4 contains the core *NbAGO5* promoter region sufficient to drive high-level expression in transgenic *N. benthamiana* under normal conditions.

### Hormone Treatments and Challenges With the Virus in Transgenic *Nicotiana benthamiana*

Since the analysis of *cis*-acting elements in the putative *NbAGO5* promoter region revealed the presence of several hormone-responsive motifs, we further tested whether the treatment of phytohormones could modulate the expression of the GUS reporter gene in transgenic plants. The result revealed that not all transgenic plant seedlings improved their GUS expression following hormone treatments (Figure 4B). ABA and SA may be important factors in affecting the GUS expression driven by PN1-PN3 regions, but only ABA was effective in PN4 plants (Figure 4B).

In order to test the influence of BaMV on the activity of *NbAGO5* promoter, transgenic *N. benthamiana* was infiltrated with *A. tumefaciens* harboring infectious construct of BaMV, or expression vector for BaMV-CP or BaMV-TGBp1. The GUS activities in the infiltrated plants were then analyzed at 3 dpi. The results of quantitative analyzes of GUS activity showed that higher *NbAGO5* promoter activity levels were observed when PN4 transgenic *N. benthamiana* was inoculated with BaMV and BaMV-TGBp1 at 3 dpi, whereas the expression of BaMV-CP did not alter *NbAGO5* promoter activity (Figure 4C). The results indicated that BaMV-TGBp1 may be the viral factor for the induction of high-level *NbAGO5* gene expression.

For testing the effect of infections by other potexviruses, PN4 transgenic *N. benthamiana* plants were agro-infiltrated with infectious constructs of various potexviruses, including BaMV, PVX, FoMV, and CymMV, and the GUS activities were analyzed at 3 dpi. The result showed that GUS activities in transgenic *N. benthamiana* were induced by the infection of all potexviruses tested, with various activities in the order of BaMV > FoMV > PVX > CymMV (Figure 4D). The result suggested that NbAGO5 may participate in the defense against potexviruses.

The above quantitative analyses of *NbAGO5* promoter activity in transgenic *N. benthamiana* PN4 line demonstrated that it was higher after potexvirus infection.

### Yeast One-Hybrid Screening of the Transcription Factor Interacting With the *NbAGO5* Promoter

Yeast One-Hybrid (Y1H) screening was used to identify possible trans-activators using a 704 bp region (PN4, positions -704 to -1 upstream of the translational initiate site ATG) of the putative *NbAGO5* promoter as the prey. The bait plasmid pHISi-PN4 was used for Y1H screening in the library of around 1,350 *Arabidopsis thaliana* TFs (Mitsuda et al., 2010). The pHISi-PN4 bait plasmid was transformed into a Y187 cell and plated on SD/-Leu-Ura for the selection of successfully co-transformed cells, which could be used for screenings of *Arabidopsis* TFs for positive interactions with the PN4 region by the addition of 75, 100, and 125 mM 3-AT. A total of 31 positive TFs, including 1, 11, and 19 with strong, weak, and very weak interactions, respectively, were obtained from this screening. We classified these candidate TFs and used them as the queries to search in *N. benthamiana* genome database by BLAST (Altschul et al., 1990) for positive ones, excluding very weak interactions (Table 1). The result revealed five TFs, including NAC, B3, C_2_H_2_ZnF, FAR1_related, and Trihelix classified as transcription factors in *A. thaliana* genome. Through homology searches in *N. benthamiana* database using BLAST, 8 homologous genes (*NAC21/22, NAC42, NAC68, NAC94, ZFP1, ZFP3, Far1-related sequence 4 isoform 3*, and *Trihelix transcription factor GT-2*) were identified in the *N. benthamiana* genome. To further verify the functions in antiviral defense, primers were designed based on the above 8 homologous genes (Supplementary Table 1), and the expression of these genes was analyzed by qRT-PCR with specific primers in healthy and BaMV-infected *N. benthamiana* leaves. The result showed that only *NbNAC42* and *NbZFP3* could be detected and isolated from health and BaMV-infected leaves of *N. benthamiana* (Supplementary Figure 1). Thus *NbNAC42* and *NbZFP3* were chosen as the main subjects for the following analyzes.

**TABLE 1:** Transcription factors binding to *NbAGO5* promoter by screening of yeast one-hybrid libraries.

Screening of Y1H libraries	Isolated TF		TF classification	Interaction strength	Homology gene in *N. benthamiana* transcriptome
	Locus	Name			
*Arabidopsis*	AT3G12910	NAC	NAC	Strong	NAC42, NAC68, NAC94
	AT3G49610	B3	B3	Weak	No
	AT5G04390	C2H2ZnF	C2H2ZnF	Weak	ZFP1, ZFP3
	AT3G10470	C2H2ZnF	C2H2ZnF	Weak	ZFP1, ZFP3
	AT2G37430	ZAT11	C2H2ZnF	Weak	ZFP1
	AT2G28200	C2H2ZnF	C2H2ZnF	Weak	ZFP1, ZFP3
	AT3G46070	C2H2ZnF	C2H2ZnF	Weak	ZFP1
	AT2G28710	C2H2ZnF	C2H2ZnF	Weak	ZFP1
	AT3G19580	ZF2	C2H2ZnF	Weak	ZFP1
	AT3G22170	CPD45	FAR1_related	Weak	Far1-related sequence 4 isoform 3
	AT3G12977	NAC1L	NAC	Weak	NAC21/22
	AT2G35640	Homeodomain-like superfamily protein	Trihelix	Weak	Trihelix transcription factor GT-2

**FIGURE 5:**
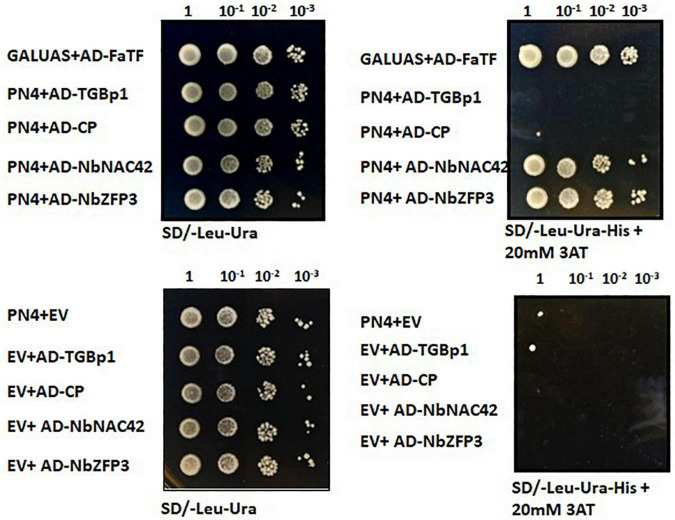
Yeast one-hybrid assay for the interaction between *NbAGO5* promoter (704 bp) and transcription factors or BaMV proteins. Yeast strain Y187 expressing PN4 (prey) was transformed with pGADT7-CP, pGADT7-TGBp1, pGADT7-NbNAC42, or pGADT7-NbZFP3 (bait). Transformed yeast cells were selected on SD minimal medium without Leu, Ura, and His but containing 20 mM 3-AT. Empty prey and bait vectors were used as negative controls. Coexpression of GALUAS along with FaTF was used as the positive control.

To further analyze the functions of NbNAC42 and NbZFP3 protein in BaMV infection, Y1H experiments were performed using the PN4 fragment of putative *NbAGO5* promoter as the bait. The result revealed that both NbNAC42 and NbZFP3 proteins could interact strongly with the PN4 region of the putative *NbAGO5* promoter, but BaMV-CP and BaMV-TGBp1 did not (Figure 5), suggesting NbNAC42 and NbZFP3 proteins may play a central role in regulating the expression of *NbAGO5*. In contrast to the result in Figure 4, which showed that BaMV-TGBp1 could induce a high-level gene expression of *NbAGO5*, the result of this Y1H experiment indicated that the up-regulation of NbAGO5 expression may be an indirect induction effect of BaMV-TGBp1, possibly through the regulation of NbNAC42 and NbZFP3 proteins, and not caused by the direct interaction between BaMV-TGBp1 and the PN4 region of the putative *NbAGO5* promoter.

### Effects of NbNAC42 and NbZFP3 on the Expression of *NbAGO5* in *Nicotiana benthamiana*

**FIGURE 6:**
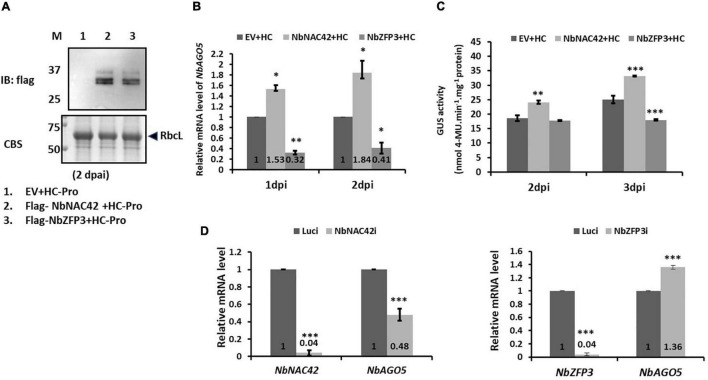
Effects of overexpression and silencing of *NbNAC42* and *NbZFP3* on *NbAGO5* levels in *N. benthamiana*. **(A)** Immunoblot analysis using anti-Flag antibody confirmed the expression of Flag-tagged NbNAC42 and NbZFP3. Total protein extracts were prepared from *N. benthamiana* leaves at 2 days post-agroinfiltration (dpai) and underwent SDS-PAGE, followed by Coomassie blue staining (CBS), anti-Flag immunoblotting. M, prestained protein markers on the left with molecular mass (in kDa). RbcL, RuBisCO large subunit is the loading control. **(B)** The expression level of *NbAGO5* was analyzed by qRT-PCR. **(C)** The effects of NbNAC42 and NbZFP3 on *PN4* activity. The GUS expression level of PN4 transgenic *N. benthamiana* plants transiently expressed *NbNAC42* and *NbZFP3* by Agrobacterium-mediated expression was analyzed at 2 and 3 dpi. **(D)** The transcript level of *NbAGO5* in *N. benthamiana* transiently knock-down the expression of *NbNAC42* and *NbZFP3*. Statistical significance was analyzed using two-tailed student’s *t*-tests; *p < 0.05; **p < 0.01; ***P < 0.001.

To verify the effect of NbNAC42 and NbZFP3 on the regulation of *NbAGO5* expression, transient over-expression, and silencing analyzes were performed. For transient expression, the coding regions of *NbNAC42* and *NbZFP3* genes were amplified, with Flag-tag, and cloned into pEpyon to generate pEpyon-NbNAC42 and pEpyon-NbZFP3. Following the verification of the constructs, the third, fourth, and fifth leaves (from the bottom to the top along the stem of seedlings) of the 30-day-old *N. benthamiana* were infiltrated with *A. tumefaciens* GV3850 cells harboring pEpyon-NbNAC42 and pEpyon-NbZFP3. Leaves were sampled for analysis at 1 and 2 dpi. The expression of Flag-tagged NbNAC42 and NbZFP3 was confirmed by immunoblot assay using an anti-Flag antibody (Figure 6A). The expression level of *NbAGO5* was then analyzed by qRT-PCR. The result revealed that mRNA levels of *NbAGO5* at 1 and 2 dpi were 1.53- and 1.84-fold higher, respectively, in leaves of plants transiently over-expressing NbNAC42 than those of the plants infiltrated with control constructs. In contrast, expression of *NbAGO5* was 0.32- and 0.41-fold lower at 1 and 2 dpi, respectively, in plants transiently expressing NbZFP3 than those in plants infiltrated with the control construct (Figure 6B). Similar trends were observed in transgenic plants harboring PN4-GUS reporter constructs. The GUS expression level at 3 dpi was 1.32-fold higher in plants transiently overexpressing NbNAC42, but 0.71-fold lower in those transiently expressing NbZFP3, as compared to the GUS expression level in plants agro-infiltrated with empty vector (EV) (Figure 6C).

For gene silencing analysis, the TRV-based VIGS system (Ratcliff et al., 2001) was used to transiently knockdown the expression of NbNAC42 and NbZFP3 in *N. benthamiana*. *NbNAC42* and *NbZFP3* mRNA levels in leaves infiltrated with TRV2-*NbNAC42* and TRV2-*NbZFP3* were both decreased to 0.04-fold of those of the negative control (Luci, leaves infiltrated with TVR2-Luc) at 10 dpi (Figure 6D). The result of the qRT-PCR revealed that the mRNA level of *NbAGO5* was decreased to 0.48-fold in NbNAC42-knockdown plants as compared to that of the negative control, but increased to 1.36-fold of that of the negative control when *NbZFP3-*knockdown plants (Figure 6D). The results of over-expression and silencing analyzes further supported the hypothesis that *NbAGO5* expression was positively and negatively associated with NbNAC42 and NbZFP3, respectively.

### Responses of NbNAC42 and NbZFP3 on Challenges of Various Viruses in *Nicotiana benthamiana*

**FIGURE 7:**
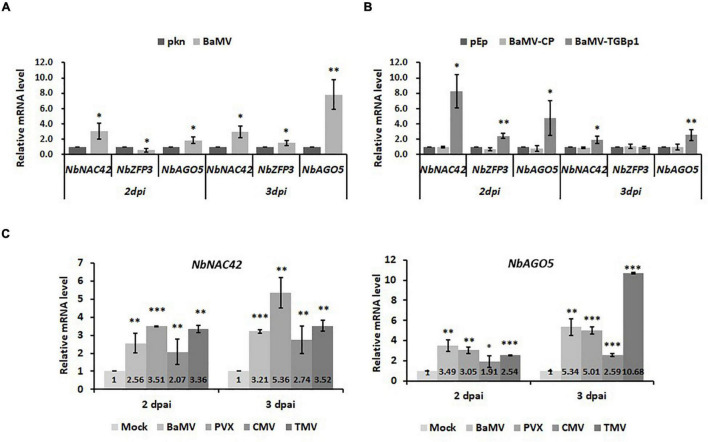
Expression of *NbNAC42* and *NbZFP3* in response to challenges of various viruses in *N. benthamiana*. The expression level of *NbAGO5, NbNAC42*, and *NbZFP3* were analyzed by qRT-PCR in the plants infiltrated with *A. tumefaciens* harboring infectious construct of BaMV **(A)**, or expression vectors for BaMV-CP or BaMV-TGBp1 **(B)**. **(C)** The mRNA level of *NbNAC42* and *NbAGO5* were analyzed by qRT-PCR in the plants infiltrated with *A. tumefaciens* harboring infectious constructs of BaMV, PVX, CMV, and TMV at 2 and 3 dpi as compared to those in control groups. Relative mRNA accumulation levels of three genes were individually shown above; numbers the compared to controls (pkn or pEpyon). Data are means ± SE from at least three independent experiments. Statistical significance was analyzed using two-tailed student’s *t*-tests; *p < 0.05; **p < 0.01; ***P < 0.001.

To verify the effects of NbNAC42 and NbZFP3 on the expression of *NbAGO5* in *N. benthamiana* infected by different viruses, the following experiments were performed. *Nicotiana benthamiana* plants were inoculated with BaMV, PVX, CMV, or TMV, or infiltrated with the constructs, pEpyon-BaMV-TGBp1, and pEpyon-BaMV-CP, for transient over-expression of BaMV-TGBp1 and -CP, respectively. Leaf samples were collected at 2 and 3 dpi and analyzed by qRT-PCR with specific primers (Supplementary Table 1). The result revealed that the mRNA expression levels of *NbNAC42* and *NbAGO5* were significantly increased following the challenge of viruses or the expression of BaMV-TGBp1 at both 2 and 3 dpi as compared to those in the control plants (Figure 7B). This result was similar in accordance with those observed in previous experiments (Figure 6B), in which the transient over-expression of NbNAC42 in *N. benthamiana* leaves led to a significant increase in the relative mRNA level of *NbAGO5*. Thus, NbNAC42 seems to play an important role in inducing *NbAGO5* expression. In contrast, the relative mRNA level of *NbZFP3* was much less than that of *NbNAC42*. The expression of *NbZFP3* in BaMV-inoculated plants was even reduced at 2 dpi (Figure 7A) but increased in the same plants at 3 dpi and in those expressing BaMV-TGBp1 (Figure 7B). This observation indicated that even if NbZFP3 may negatively regulate the expression of *NbAGO5* (Figure 6D), the expression level and speed of NbZFP3 might not be sufficient to antagonize the positive effect of NbNAC42 on *NbAGO5* expression in *N. benthamiana*. In addition, it was also found that the mRNA level of *NbNAC42* and *NbAGO5* were significantly increased in response to the infections of BaMV, PVX, CMV, and TMV at 2 and 3 dpi as compared to those in control groups (Figure 7C). The result suggested that the NbNAC42 may serve as the sensor of the antiviral surveillance system in *N. benthamiana* by interacting with viral factors (such as BaMV-TGBp1), and subsequently activating the expression of NbAGO5 which in turn participates in the RNA silencing mechanism in the defense against different viruses.

## Discussion

**FIGURE 8:**
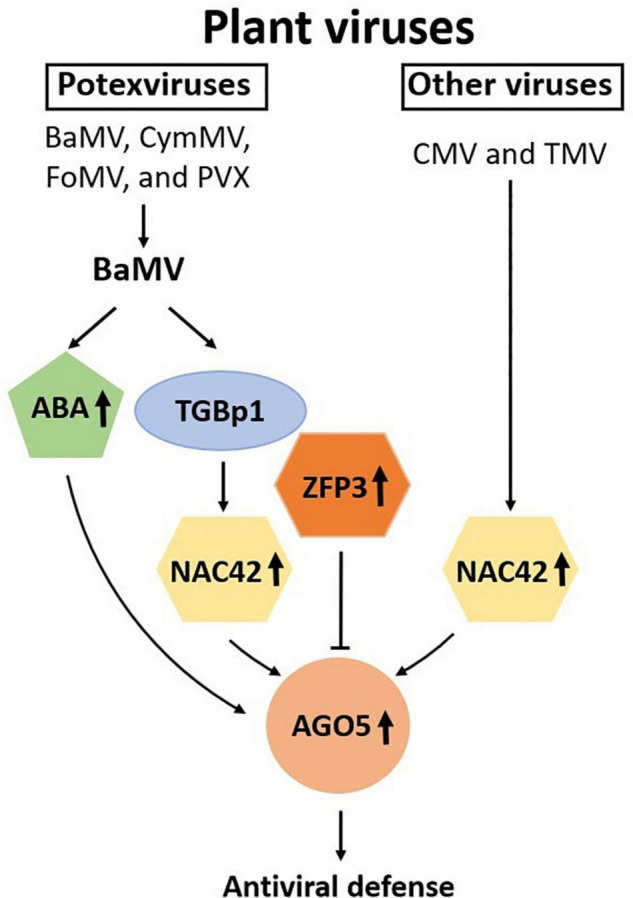
Schematic representation of the involvement of NAC and ZFP transcription factors and ABA in the regulation of *NbAGO5* expression in response to virus infection. Plant argonautes (AGOs) play an important role in antiviral defense. The expression of *NbAGO5* gene is strongly induced after virus infection in *N. benthamiana*. NbNAC42 and NbZFP3 positively and negatively regulate the expression of *NbAGO5*, respectively. The plant hormone ABA, whose biosynthesis has been shown to be activated by BaMV infection (Alazem et al., 2014), is also involved in the induction of *NbAGO5* promoter activity. TGBp1, a BaMV-encoded protein, may regulate *NbAGO5* expression and plant defense response through activation of the *NbNAC42* expression.

Plant AGO proteins are guide-dependent nucleases involved in important functions, including developmental processes and stress responses against biological or abiotic stresses (Carbonell, 2017). For defense against plant viruses, it is known that different AGO proteins may be recruited in a virus-specific manner. However, the mechanisms for the specific induction of unique AGO proteins have not been explored extensively. In this study, we have attempted to elucidate the chain of command, at least in part, for the defense against specific viruses in *N. benthamiana*. The result revealed that BaMV encoded protein, TGBp1, may trigger the defense response by inducing the expression of NbNAC42, which in turn enhanced the expression of *NbAGO5* and the subsequent antiviral defense through the RNA silencing mechanism. To counteract the host defense system, BaMV-TGBp1 may have evolved the ability to induce the expression of NbZFP3, which suppressed the expression of *NbAGO5*, as depicted in the proposed model shown in Figure 8. However, the level of NbZFP3 enhancement might not be sufficient to suppress the RNA silencing mechanism of the host against the invading virus.

Previously, we have found that an AGO5 family protein of *Phalaenopsis aphrodite*, PaAGO5b, is the only AGO proteins highly induced following the infection of *Cymbidium mosaic virus* and *Odontoglossum ringspot virus*, and we also demonstrated that PaAGO5b plays an important role in the defense against these viruses. However, the mechanism for the elicitation of PaAGO5b expression remains to be investigated. Here, among the *N. benthamiana* AGO family proteins tested, *NbAGO5* was also found to be highly induced in *N. benthamiana* infected by BaMV. We have characterized the putative promoter of *NbAGO5* to understand its expression patterns in response to virus infection and identified two TFs, namely NbNAC42 and NbZFP3, for the modulation of *NbAGO5* expression. The core promoter region of *NbAGO5* was mapped to segment PN4, a 704 bp segment (nucleotide positions -704 to −1, upstream of the translational initiate codon of the *NbAGO5* gene) which was sufficient to drive high-level expression under the induction of virus challenges. We have found that PN4 is the key region required for response to virus infections. In *A. thaliana*, AtAGO5 has been found to be expressed in meristems (Tucker et al., 2012) and affect the megagametogenesis process (Mi et al., 2008; Tucker et al., 2012). In *N. benthamiana*, GUS histochemical staining analysis revealed that GUS was highly expressed in both flowers and seeds of transgenic plants (Figure 3), suggesting the involvement of *NbAGO5* during the stages of pollination and reproduction. This observation is in accordance with the finding that the putative *NbAGO5* promoter region contains a CAT-BOX element that has a *cis*-acting regulatory function related to meristem expression.

In addition, two types of NAC protein binding sites with the core CACG and CATGTG elements (Tran et al., 2004; He et al., 2005) were identified in the putative *NbAGO5* promoter region. We also confirmed in Y1H experiments that NbNAC42 has a strong interaction with the putative *NbAGO5* promoter region. NAC-TFs are named after three functional NAC domain-containing genes: *no apical meristem* (NAM) (Souer et al., 1996), *Arabidopsis thaliana Transcription Activator Factor 1/2* (ATAF1/2), and *cup-shaped cotyledon* (CUC) (Aida et al., 1997). NAC proteins are divided into two structural regions: one N-terminal DNA-binding domain of 151–159 amino acids and C-terminal transcriptional regulatory region that are highly divergent (Aida et al., 1997; Kikuchi et al., 2000; Jensen et al., 2010). Comparative genomic and gene functional analyzes were used to divide NAC proteins into six major orthologous groups (Groups I–VI) using 2,106 non-redundant sequences from 24 different green plant species (Pereira-Santana et al., 2015). NbNAC42 is classified as Group IV. The members of NAC group IV perform a variety of functions, such as the regulation of responses in drought, salinity (Tak et al., 2017), longevity (Wu et al., 2012), and resistance against the virus (Thirumalaikumar et al., 2018), as demonstrated for JUNGBRUNNEN1 (JUB1 or NAC042). Although many NACs have been characterized as having anti-viral ability (Ren et al., 2000; Selth et al., 2005; Donze et al., 2014; Huang et al., 2017a; Sun et al., 2019), the activation mechanisms and the downstream targets for NAC proteins remain largely unknown. The results of this study revealed that the expression of NbNAC42 could be enhanced by BaMV-TGBp1, and that NbNAC42 achieves the antiviral effect by up-regulating the expression of *NbAGO5*, which is involved in the RNA silencing-mediated antiviral defense system.

Zinc finger proteins have been shown to be regulators of abiotic stress responses in plants (Han et al., 2020) and divided into subgroups based on the order of Cys and His residues in their secondary structures, such as Cys2/His2-type (C_2_H_2_), C_3_H, C_3_HC_4_, C_2_HC_5_, C_4_HC_3_, C_2_HC, C4, C6, and C8 (Miller et al., 1985; Klug, 2010; Han et al., 2014). NbZFP3 is classified as C2H2-type, which could directly target genes involved in hormone signal transduction (Kodaira et al., 2011) and regulate the responses to stresses of salt (Huang et al., 2007; Ma et al., 2016), osmotic pressure (Sun et al., 2010; Han et al., 2019), reactive oxygen species (Rizhsky et al., 2004; Vogel et al., 2005; Sun et al., 2010; Liu et al., 2017), and cold (Vogel et al., 2005; Kim et al., 2016) stress. C_2_H_2_-type ZFPs have the Ethylene-responsive element-binding factor-associated amphiphilic repression (EAR) motif [with the conserved amino acid sequence of *^L^*/_*F*_DLN *^L^*/_*F(x)*_P] in the C-terminal region (Ohta et al., 2001). The EAR motif has been shown to lower the transcriptional activity of the reporter gene and other TFs (Kazan, 2006). NbZFP3 has an EAR motif starting at amino acid position 312, which specifically binds to the A(G/C)T repeat sequences (Sakamoto et al., 2004) in their target promoters. The negative correlation between the expression of *NbAGO5* and NbZFP3 could be due to the binding of NbZFP3 to the *NbAGO5* promoter through the EAR motif, which in turn repressed the expression of *NbAGO5*. This may serve as the strategy of the invading virus to suppress the defense system of the host. However, it should be noted that the induction level of NbZFP3 by the infection of BaMV or the expression of TGBp1 might not be high enough to counteract the enhancement effect on *NbAGO5* expression by NbNAC42, as shown in Figure 7B.

It is worth noting that the JA-responsive transcription factor, JAMYB, in rice has been shown to activate the expression of *AGO18*, a core RNA silencing component, by directly binding to its promoter, thus enhancing the antiviral defense (Yang et al., 2020). It was shown that the activation of JAMYB expression is mainly by the CP of the rice stripe virus, which stimulates the accumulation of JA. In comparison, the results of our work showed that the *AGO5* promoter (PN4) contains an MYB binding site; however, there appeared to be no interaction with MYB-type TFs in the screenings of the *Arabidopsis* TFs library (Table 1). Furthermore, infecting *N. benthamiana* with BaMV or CMV increased ABA levels and activated the SA and ABA pathways, reversing the antagonistic relationship between these two pathways (Alazem et al., 2014). Therefore, this means that the AGO family with antiviral ability is diversified in the mechanisms of initiating antiviral defense. This also implies that, as plants evolve, different defense mechanisms could be activated in response to different virus threats. In Figure 7C, the result revealed that potexviruses may trigger the defense response by inducing the expression of *NbNAC42*. For BaMV infections, we showed that BaMV- TGBp1 may serve as an activator for *NbNAC42* expression (Figure 7B). We also tested the effect of other viruses on the expression of *NbNAC42*, such as CMV and TMV (Figure 7C). Thus, the expression of *NbNAC42* may be sensitive to various virus infections, and in turn, may enhance the expression of *NbAGO5* to defend against viruses.

In addition to the contribution to the understanding of antiviral defense mechanisms, the virus-inducible *NbAGO5* promoter may have practical applications in plant biotechnology. Currently, the commonly used promoters for the expression of foreign genes in plants are constituent promoters, such as the CaMV35S promoter and the maize ubiquitin promoter. These constituent promoters are usually active in almost all tissues and developmental stages of plants, which might result in excessive energy loss and possible morphological and physiological dysfunction. In contrast, inducible or tissue-specific promoters may regulate the expression of target genes in particular conditions or tissues, which is more energy conservative and easier for the maintenance of normal physiological functions of plants. In this study, we have demonstrated that the *NbAGO5* promoter could be positively and negatively regulated by NbNAC42 and NbZFP3, respectively, in response to virus infections or hormone treatment (such as ABA, Figure 4B). Thus, this viral stress-inducible promoter would be an ideal candidate for the controlled overexpression of foreign proteins for academic or industrial purposes.

Overall, this study demonstrated the involvement of NbAGO5 in the defense against specific viruses in *N. benthamiana*, and revealed the underlying mechanism for the modulation of *NbAGO5* expression by two antagonizing TFs, NbNAC42, and NbZFP3, in response to specific virus infections or the presence of specific viral protein, such as BaMV-TGBp1. These findings provided further insights into the chain of command in the antiviral defense system in plants and may be further applied in the fields of crop improvement or plant biotechnology.

## Data Availability Statement

The original contributions presented in this study are included in the article/Supplementary Material, further inquiries can be directed to the corresponding author.

## Author Contributions

Y-DK and KV performed the isolation and functional analysis of the NbAGO5 promoter. C-MY and NM performed the yeast one hybrid screening analysis. Y-DK and Y-WH revealed the underlying mechanism for the modulation of *NbAGO5* expression by two antagonizing TFs in the defense against viruses. Y-DK drafted the manuscript. C-CH, C-MY, N-SL, and Y-HH helped to draft the article structure of the manuscript. Y-HH supervised the entire study and organized the participation of each lab participant. All authors read and approved the manuscript.

## Conflict of Interest

The authors declare that the research was conducted in the absence of any commercial or financial relationships that could be construed as a potential conflict of interest.

## Publisher’s Note

All claims expressed in this article are solely those of the authors and do not necessarily represent those of their affiliated organizations, or those of the publisher, the editors and the reviewers. Any product that may be evaluated in this article, or claim that may be made by its manufacturer, is not guaranteed or endorsed by the publisher.

## Footnotes

1
https://benthgenome.qut.edu.au

2
http://bioinformatics.psb.ugent.be/webtools/plantcare/html/

3
https://solgenomics.net/tools/blast

4
https://solgenomics.net/organism/Nicotiana_tabacum/genome
