# Supplementary material for: NbNAC42 and NbZFP3 Transcription Factors Regulate the Virus Inducible NbAGO5 Promoter in Nicotiana benthamiana
Source: Front Plant Sci. 2022 Jun 23;13:924482. doi: 10.3389/fpls.2022.924482 (PMC9261433; doi:10.3389/fpls.2022.924482)
Supplement: Supplementary file 1 [file Presentation_1.PPTX]

## Slide 1
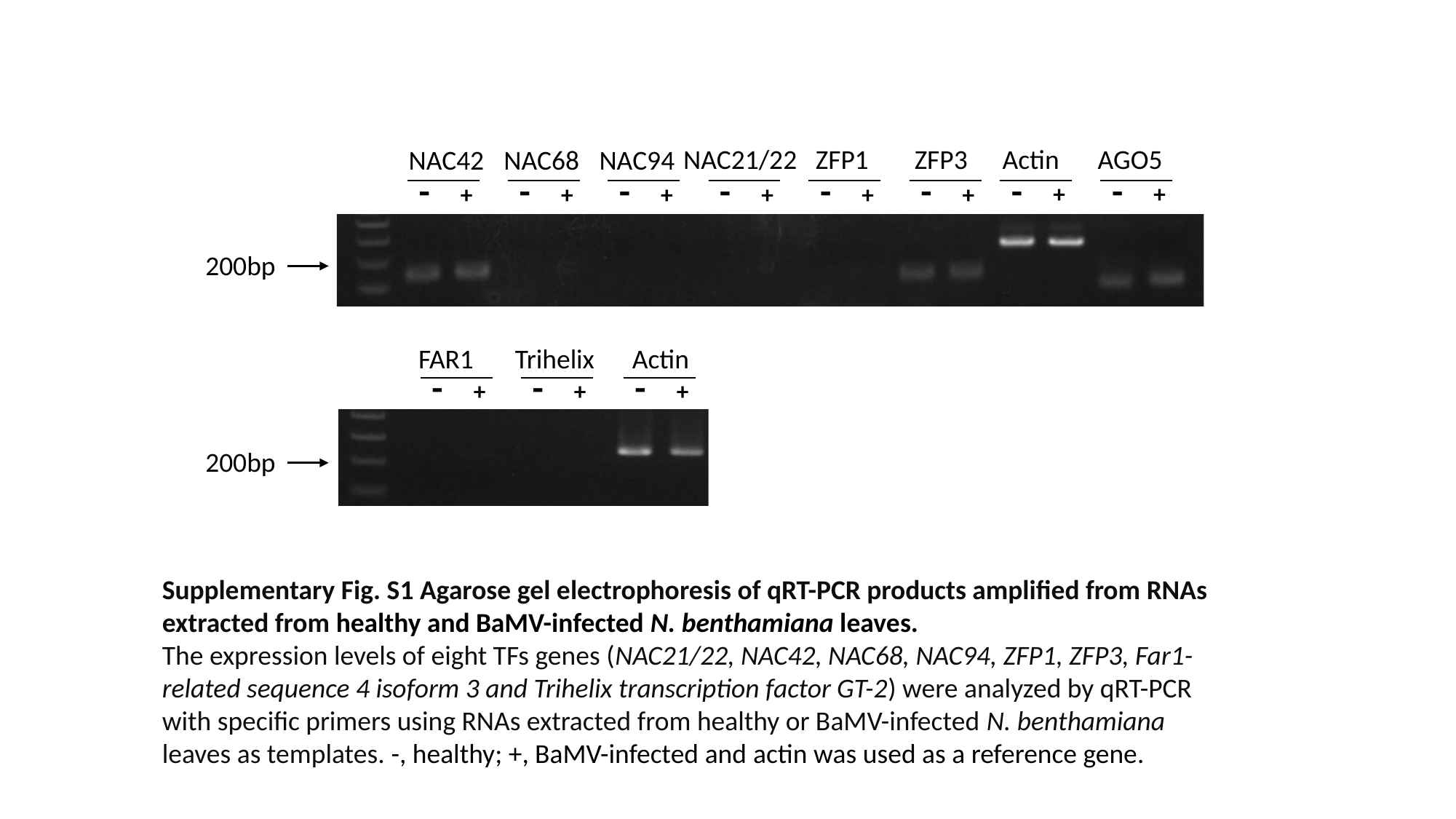

NAC21/22
ZFP3
Actin
AGO5
ZFP1
NAC42
NAC68
NAC94
-
-
-
-
-
-
-
-
+
+
+
+
+
+
+
+
200bp
FAR1
Trihelix
Actin
-
-
-
+
+
+
200bp
Supplementary Fig. S1 Agarose gel electrophoresis of qRT-PCR products amplified from RNAs extracted from healthy and BaMV-infected N. benthamiana leaves.
The expression levels of eight TFs genes (NAC21/22, NAC42, NAC68, NAC94, ZFP1, ZFP3, Far1-related sequence 4 isoform 3 and Trihelix transcription factor GT-2) were analyzed by qRT-PCR with specific primers using RNAs extracted from healthy or BaMV-infected N. benthamiana leaves as templates. -, healthy; +, BaMV-infected and actin was used as a reference gene.
